# The regulatory landscape for extracellular vesicle therapies: Australian context and future directions

**DOI:** 10.20517/evcna.2025.129

**Published:** 2026-02-27

**Authors:** Christopher Rudge, Reeve McClelland, Wojciech Chrzanowski, Ryan L. Davis

**Affiliations:** ^1^Sydney Health Law, Sydney Law School, The University of Sydney, Camperdown 2006, Australia.; ^2^Sydney Pharmacy School, Faculty of Medicine and Health, The University of Sydney, Camperdown 2006, Australia.; ^3^Division of Biomedical Engineering, Department of Materials Science and Engineering, Uppsala University, 75105 Uppsala, Sweden.; ^4^School of Medical Sciences, Faculty of Medicine and Health, The University of Sydney, Camperdown 2006, Australia.; ^5^Kolling Institute, University of Sydney and Northern Sydney Local Health District, St Leonards 2065, Australia.

**Keywords:** Extracellular vesicles, Australian therapeutic goods regulation, precision medicine variability, innovative medicines regulation

## Abstract

Extracellular vesicles (EVs) are emerging as promising tools for regenerative medicine and drug delivery, offering unique therapeutic advantages. However, their clinical translation - in Australia and globally - faces persistent challenges. These are commonly framed as technical issues stemming from inherent EV variability and the absence of standardized potency assays. While no EV-based therapeutic has yet received full market approval from any major regulatory agency, this Perspective argues that the barriers to translation are not solely technical but reflect limitations within current regulatory frameworks. In Australia, the Therapeutic Goods Administration (TGA) requires biological products to be included on the Australian Register of Therapeutic Goods (ARTG) before supply to market. However, several alternative regulatory pathways exist that can facilitate clinical access to “unapproved” products. Through analysis of these pathways, and comparison with international approaches, this Perspective highlights how regulatory inflexibility may be as significant a barrier to translating EV medicines as the technical difficulties themselves. Drawing on insights from governmental inquiries into the approval and subsidization of emerging medicines, the Perspective calls for reform of Australia’s regulatory systems - including development of EV-specific guidance and policy that introduces adaptive assessment pathways - to better support the safe and timely integration of novel biotechnologies.

## INTRODUCTION

Extracellular vesicles (EVs) have been described as a “rising star for therapeutics and drug delivery”^[1]^, playing targetable roles in cancers and neurodegenerative disorders while possessing programmable, biologically derived drug-carrying capabilities^[2,3]^. The field has witnessed substantial growth, with over 90 clinical trials registered for therapeutic applications as of 2024, predominantly investigating mesenchymal stem cell-derived EVs for conditions ranging from respiratory illness to stroke^[4]^. While these nanovesicles possess ideal properties for cell-free regenerative medicine (biocompatibility and minimal immunogenicity)^[5]^, they face critical barriers to clinical translation: “batch-to-batch variability, donor material diversity, and the lack of standardized potency testing”^[6]^, issues that have historically plagued precision medicines more broadly. Notably, no EV-based therapeutic has yet received full market approval from the Food and Drug Administration (FDA), European Medicines Agency (EMA), or Therapeutic Goods Administration (TGA) - though the FDA’s November 2024 approval of Ryoncil (remestemcel-L), a mesenchymal stromal cell therapy with EV-mediated mechanisms, marks a significant milestone^[6]^. These difficulties have prompted calls for “clear and concise regulations” for EV-based interventions^[7]^. However, focusing on conventional market authorization as the sole means of clinically translating EVs - via inclusion on the Australian Register of Therapeutic Goods (ARTG) - adopts an overly narrow view.

The regulatory framework in Australia, administered by the TGA, provides multiple pathways to clinical translation. While ARTG inclusion facilitates “commercial supply of therapeutic goods”^[8]^, alternative access schemes and classification rules offer potential routes for EV-based therapies that do not demand the evidence required for full ARTG registration of high-risk biologicals. Given these alternatives, the question arises: are clinical translation challenges particular to EV medicines, or is the regulatory system fundamentally unsuitable for precision medicines’ inherent variability? A 2021 parliamentary inquiry (the New Frontier Report) suggests the latter^[9]^. The difficulties faced by EVs may stem not only from technical barriers but from regulatory inadequacies relating to dated legal frameworks. This Perspective examines these inadequacies, analyzes alternative pathways and classification possibilities, compares international approaches, and considers whether policy revision is needed. It also addresses systemic questions raised by the New Frontier Report and recent health technology assessment (HTA) reviews^[10]^.

## REGISTERING EV BIOLOGICALS ON THE ARTG

The conventional method of supplying therapeutic products in Australia is ARTG inclusion - a database listing all therapeutic goods “approved for supply” by the TGA^[8]^. Despite 49 biological products on the ARTG as of April 2025^[11]^, none are EV medicines.

### The challenge of variability

Key technical hurdles include scalability and standardization of EV production, lack of appropriate potency assays, and inefficient targeted delivery^[5,12]^. Inherent biological variability - from diverse primary stem cell sources and in vitro expansion effects - results in heterogeneous therapeutic EV populations^[6]^.

This heterogeneity poses significant challenges under the Regulatory Framework for Biologicals (RFB), set out in the *Therapeutic Goods Act 1989* (Cth) (TG Act) and *Therapeutic Goods Regulations 1990* (Cth). Biologicals, defined in section 32A as goods comprising, containing or derived from human cells or tissues, must be supported by rigorous evidence demonstrating consistent manufacturing, characterization, safety, and efficacy^[13]^. The lack of standardized potency assays makes meeting these requirements difficult. This issue extends beyond Australia: the FDA similarly cited “chemistry, manufacturing and control (CMC) issues” when initially denying remestemcel-L approval in August 2023^[14]^.

However, variability challenges are not solely attributable to biological complexity or regulatory stringency. Systematic reviews reveal substantial reporting gaps: only 12.1% of EV clinical trials report isolation methodology, and merely 36.1% specify characterization methods^[4]^. The Minimal Information for Studies of Extracellular Vesicles (MISEV) guidelines, published to standardize EV characterization, have been inconsistently adopted^[15]^. These deficits impede reproducibility, regulatory assessment and inter-study comparison, undermining the evidence base for approval decisions. Addressing variability therefore requires both regulatory adaptation and improved standardization within research and manufacturing communities.

### The challenge of classification

The RFB classifies biologicals from Class 1 (lowest risk) to Class 4 (highest risk), with each class bearing different evidentiary burdens^[16,17]^. The regulatory classification of EV-based products within this framework has been previously examined, with EVs derived from non-pluripotent stem cells likely classified as Class 3 biologicals requiring detailed evidence dossiers^[18]^. The TGA’s Technical Requirements^[19,20]^ mandate that Class 2 biologicals demonstrate quality and manufacturing controls, with efficacy justified by scientific literature rather than product-specific trials. Class 3 and 4 biologicals require comprehensive non-clinical data (in vitro and animal studies) and clinical data (dose determination, safety and efficacy trials, and biovigilance plans).

This difference points to classification’s critical impact. Unmodified EVs argued as Class 2 biologicals (minimal manipulation, homologous use) face significantly lower evidentiary hurdles. Modified EVs or those for non-homologous uses - likely Class 3 or 4 - must satisfy demanding evidence requirements. These challenges have contributed to the absence of EV medicines on the ARTG and the lack of “widespread clinical approval” globally^[12]^.

## ALTERNATIVE ACCESS PATHWAYS: THE SAS, APS, AND SPECIFIC EXEMPTIONS

**Figure 1:**
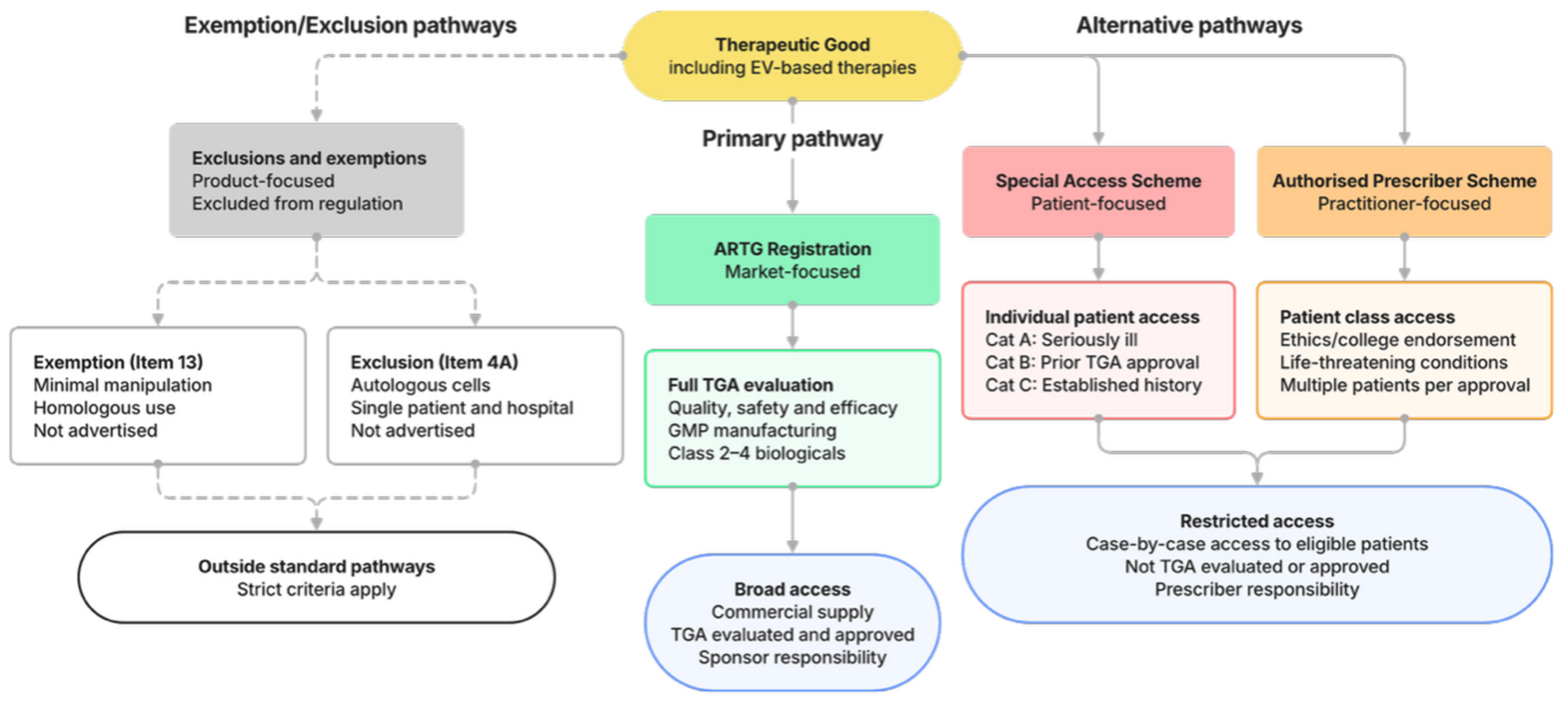
Regulatory pathways for therapeutic goods in Australia, including EV-based therapies. Products may access patients through ARTG registration (approved pathway) or the Special Access Scheme and Authorized Prescriber pathways (unapproved). Certain biologicals may also fall outside standard TGA oversight through exclusions (Item 4A) or exemptions (Item 13), subject to strict criteria. ARTG: Australian Register of Therapeutic Goods; EV: extracellular vesicle; TGA: Therapeutic Goods Administration.

The Australian framework provides alternative mechanisms permitting access to therapeutic goods without ARTG approval [Figure 1]. These include the Special Access Scheme (SAS), the Authorized Prescriber Scheme (APS), and specific exemptions or exclusions. These pathways operate under different evidentiary assumptions and may help address some challenges associated with the biologicals framework. However, they are designed for individual patient access or specific clinical contexts - exceptions to market authorization, not scalable pathways for commercial translation. In the absence of permissive and inclusive policy, it is likely that attempts will be made to use these pathways to facilitate access to EV medicines.

### The SAS

The SAS, established under section 19(1) of the TG Act, enables practitioners to access unapproved goods for individual patients on a case-by-case basis. However, the legislation requires practitioners to first consider ARTG-listed options^[21]^. If practitioners use the SAS to prescribe an unapproved good, the prescriber will assume responsibility for that clinical decision.

SAS Category A (“seriously ill patient pathway”) permits access for patients facing likely death within months^[22]^, requiring only informed consent and TGA notification within 28 days of administration^[23]^. Category B (“non-seriously ill pathway”) requires prior TGA approval with clinical justification^[21]^. Category C (“established history of use”) covers specific unapproved but previously used products and only requires notification to the TGA within 28 days of administration; as of April 2025, no EV medicines are listed^[24]^.

For EVs, Category B appears most likely, accommodating novel therapies where standard treatments are unsuitable. However, prescribers must source products independently, and unapproved products are generally not subsidized by the Pharmaceutical Benefits Scheme (PBS), so costs are passed to patients^[25]^.

### The APS

The APS [TG Act section 19(5)] allows practitioners to supply specific unapproved products to patient classes without case-by-case approval^[26]^. For biologicals specifically, TG Act section 32CM(1) permits the TGA to authorize practitioners to supply certain biologicals to specified patient classes, requiring human research and ethics committee (HREC) endorsement and approval. This pathway is limited to patients with life-threatening or serious conditions, and has facilitated access to novel therapeutics^[27]^. Further, section 32CM(7A) allows the Minister to authorize entire practitioner classes to be included as an APS class via legislative instrument.

### Leveraging regulatory exceptions

The TG Act provides circumstances where human cell or tissue (HCT) products may be administered outside standard requirements through certain exclusions^[28]^ or exemptions^[29]^.

Item 4A of the Excluded Goods Determination 2018 excludes HCT goods that are: (i) derived from a patient’s own cells under practitioner care; (ii) manufactured under practitioner supervision in hospital; (iii) used solely for that patient; and (iv) not advertised^[30]^.

Item 13 of Schedule 5A exempts HCT products meeting stricter criteria: autologous, practitioner-supervised manufacture, single homologous use indication, single clinical procedure, and minimal manipulation^[31,32]^. Whether EV extraction constitutes “minimal manipulation” and defining “homologous use” for EVs remain interpretive challenges requiring further policy development.

### Limitations of alternative pathways

While these pathways offer routes for specific autologous EV applications to bypass pre-market assessment, their stringent conditions limit broader applicability. The SAS and APS are patient-access mechanisms, not commercialization pathways - they shift regulatory burden to practitioner responsibility without resolving challenges of demonstrating consistent manufacturing and efficacy across populations.

Moreover, reduced oversight raises patient safety considerations. Practitioners assume responsibility without TGA evaluation. While appropriate for seriously ill patients lacking alternatives, this creates potential for poorly characterized products to reach patients without being subject to regulatory scrutiny (via the ARTG). These pathways represent a trade-off between access and assurance - justified in specific circumstances but no substitute for systemic reform.

## THE POLICY QUESTION: SHOULD EVs BE SPECIFIED AS “NOT BIOLOGICALS”?

A fundamental question is whether EVs should be regulated as biologicals at all. TG Act section 32A(3) empowers the Secretary of the Department of Health, Disability and Ageing to determine that certain products are not biologicals. The Therapeutic Goods (Biologicals - Specified Things) Instrument 2021 currently specifies several such things in Schedule 2, including haematopoietic progenitor cells, *in vitro* diagnostic (IVD) medical devices, and “biological medicines” (including blood components).

Could, or should, EVs be included? As with specified “biological medicines” (e.g., plasma derivatives and purified proteins), most EVs are non-viable components derived from biological sources. Excluding non-viable EVs from regulation as biologicals could shift the focus from cell-centric concepts, such as homologous use, toward a medicines-based paradigm that emphasizes manufacturing consistency, characterization, purity, and pharmacological effects - an approach particularly appropriate for unmodified EVs.

However, reclassification would shift regulatory scrutiny, not eliminate it. EVs regulated as medicines would face different but not lesser requirements: demonstration of quality, safety, and efficacy through clinical trials, with Good Manufacturing Practice (GMP) standards. The question is whether this framework better suits the development of EVs as medicines, not whether it represents an easier pathway to patient access.

EVs differ from current biological medicines in complexity and multifaceted mechanisms, with “more research... needed to unravel the distinct mechanisms of various EV subpopulations”^[33]^. Their cellular origin - mesenchymal stem/stromal cells (MSCs), immune cells, blood cells, or neural cells^[34]^ - and complex cargoes (engineered proteins, lipids, nucleic acids^[35]^) raise unique safety and efficacy considerations^[36]^.

A further complexity is that cargo-loaded EVs might be regulated as the drug itself, with EVs serving as delivery excipient. However, EVs differ from synthetic carriers through biological origin, inherent bioactivity, and potential immunogenicity. Whether EVs would be regulated as medicines or delivery vehicles remains an open question shaping evidentiary requirements.

The risk of premature reclassification is creating regulatory gaps rather than closing them. If EVs were specified as “not biologicals” without corresponding EV-specific guidance within the medicines framework, the result could be increased uncertainty. The policy question is not simply whether EVs should exit the biologicals framework, but whether any alternative framework could better accommodate these products while maintaining patient protections.

## INTERNATIONAL CONTEXT: LESSONS FOR AUSTRALIAN REFORM

The challenges facing EV translation in Australia are instructive when viewed internationally. As the New Frontier Report observed, Australia’s regulatory system requires “more flexible pathways to enable [the] system to keep pace with medical and technological advances”^[9]^. International experience demonstrates that such flexibility is achievable without compromising patient safety.

The TGA’s current approach operates within the broader biologicals framework, with classification determined by product characteristics rather than EV-specific guidance. This contrasts with several comparable jurisdictions. South Korea’s Ministry of Food and Drug Safety has published dedicated EV therapeutic guidelines, recently authorizing a Phase 1b stroke therapy trial^[37]^. Taiwan’s Regenerative Medicine Act explicitly encompasses EVs, with human-derived EVs approved for cosmetic applications^[37]^. Japan’s dual-track Pharmaceuticals and Medical Devices (PMD) Act system permits conditional, time-limited marketing authorizations - balancing early access against ongoing safety evaluation^[37]^.

The FDA regulates EVs as biologics requiring Investigational New Drug (IND) applications and Biologics License Applications (BLAs), with no EV therapeutic yet approved. Nonetheless, Breakthrough Device designations have been granted to select EV diagnostics, including ExoDx Prostate^[38]^. The EMA classifies EV therapies as Advanced Therapy Medicinal Products (ATMPs) when cargo exerts physiological effects, triggering Committee for Advanced Therapies (CAT) evaluation^[37]^. Both agencies have established expedited pathways accommodating appropriately characterized innovative products.

Two regulatory strategies emerge: classification by cargo composition and physiological effects (FDA/EMA), versus classification by manufacturing source and methodology (Asian jurisdictions)^[37]^. Australia aligns with the former. However, what distinguishes the TGA is the absence of guidance assisting sponsors with EV-specific applications. As previously analyzed, the TGA’s Priority Pathway and provisional approval mechanisms offer theoretical expedited routes^[39]^, but their effective utilization requires clearer regulatory signaling.

The EV field itself contributes to regulatory uncertainty. As noted above, substantial methodological reporting gaps persist^[4]^. While meta-analyses demonstrate favorable safety (serious adverse events in 0.7% of 335 patients across 21 studies)^[40]^, substantial heterogeneity persists in manufacturing, study design, and adverse event reporting. These standardization deficits reflect research community gaps complicating proportionate regulatory development.

This dual challenge - regulatory inflexibility compounded by field-wide standardization gaps - underscores why the New Frontier Report’s recommendations remain pertinent (discussed below). The proposed Center for Precision Medicine and Rare Diseases (discussed below) could develop EV-specific guidance informed by international precedents while promoting standardized practices. South Korea and Taiwan demonstrate that dedicated frameworks are achievable; FDA Breakthrough designations show that expedited pathways can coexist with rigorous oversight. Australia does not need to choose between innovation and safety - but achieving both requires rapid regulatory evolution and a more dynamic, agile approach.

## PRODUCT CHALLENGES VERSUS SYSTEMIC REFORM: THE NEW FRONTIER REPORT AND HTA REVIEW PERSPECTIVES

The multiple pathways to clinical translation may reflect two opposing propositions. The sympathetic view holds that Australia’s framework accommodates variable biological medicines through market-focused (ARTG), patient-focused (SAS), practitioner-focused (APS), and product-focused (exemptions) pathways. The alternative, expressed in the New Frontier Report, is that the numerous conditional pathways indicate framework inadequacy for precision medicine. The Report identified a “significant challenge” as the need to “establish more flexible pathways to enable [the] system to keep pace with medical and technological advances”^[9]^.

### The New Frontier Report

Recommendation 1 sought a Center for Precision Medicine and Rare Diseases providing “timely access to new drugs and novel medical technologies” and advising on “a dedicated regulatory HTA pathway for cell and gene technologies”^[9]^. Recommendation 2 sought HTA simplification to “establish a clear and certain pathway” for such technologies^[9]^. These signals recognize that existing frameworks may be inappropriate for innovative biologicals.

Recommendation 30’s emphasis on patient access, streamlined processes, real-world evidence, and revised valuation methods^[9]^ challenges the premise that product variability is the sole translation barrier. A Center for Precision Medicine and Rare Diseases would address the need for specialized expertise and adaptive assessment paradigms. Indeed, the government’s acceptance of these recommendations^[41]^ validates that analysis.

### HTA perspectives

The HTA Policy and Methods Review^[42]^ confirmed that “High-Cost Highly Specialised Technologies” funding pathways - analogous to complex EV medicines - require development, with current arrangements found to be “not effective” in managing uncertainties^[42]^.

A companion paper on Emerging Health Technologies^[43]^ identified the primary challenge as lack of high-quality data from trial designs adapted for small populations and shorter durations. These evidence gaps impede Pharmaceutical Benefits Advisory Committee (PBAC) assessments of comparative effectiveness and cost-effectiveness - core requirements for PBS listing.

The variability challenge therefore translates directly into evidentiary uncertainty posing substantial HTA barriers. The HTA process represents a bottleneck demanding either exceptionally robust evidence or evolution in assessment methodologies.

## CONCLUSION: CHARTING A COURSE FOR EVs IN AUSTRALIA

The path toward EV clinical translation in Australia extends beyond overcoming technical variability and standardization challenges. These are critical barriers for ARTG authorization, particularly for modified EVs classified as Class 3 or 4, and present substantial HTA challenges for PBS access.

Alternative pathways - SAS, APS, exclusions, exemptions - operate under distinct evidentiary assumptions but are access exceptions, not scalable commercial solutions. Whether EVs should be specified as “not biologicals” remains open; any reclassification would shift rather than eliminate scrutiny.

This analysis, informed by the New Frontier Report and HTA Review, suggests EV difficulties reflect not only product-specific hurdles but constraints of current systems developed for different therapeutic modalities. International experience - particularly South Korea and Taiwan - demonstrates that adaptive frameworks are achievable and should be dynamically developed for the Australian setting.

Successfully translating EV therapies requires concrete reform. First, the TGA should develop EV-specific guidance clarifying classification criteria, characterization expectations, and evidentiary approaches. Such guidance, informed by international precedents and MISEV, would reduce sponsor uncertainty and facilitate appropriate pathways. Second, implementation of the Center for Precision Medicine and Rare Diseases should be accelerated, with explicit EV inclusion. This Center could provide specialized expertise for assessing inherently variable products. Third, research and manufacturing communities must improve standardized characterization and reporting practices. Regulatory reform alone cannot resolve translation challenges if the evidence base remains compromised by methodological inconsistency.

These reforms require coordinated action from regulators, policymakers, researchers, and industry. The objective: a regulatory framework that enables safe innovation while accommodating the characteristics of precision medicine, aligned globally to ensure equitable access to EV medicine. Australia has the scientific expertise and regulatory infrastructure to lead; what is required is policy commitment to adapt existing frameworks for emerging therapeutic modalities.

## DECLARATIONS

### Copyright

© The Author(s) 2026.

## References

[1] Du S, Guan Y, Xie A (2023). Extracellular vesicles: a rising star for therapeutics and drug delivery. J Nanobiotechnology.

[2] Kumar MA, Baba SK, Sadida HQ (2024). Extracellular vesicles as tools and targets in therapy for diseases. Signal Transduct Target Ther.

[3] Jay SM (2025). Addressing barriers to clinical translation of extracellular vesicle therapeutics. Mol Ther.

[4] Mizenko RR, Feaver M, Bozkurt BT (2024). A critical systematic review of extracellular vesicle clinical trials. J Extracell Vesicles.

[5] Moghassemi S, Dadashzadeh A, Sousa MJ (2024). Extracellular vesicles in nanomedicine and regenerative medicine: a review over the last decade. Bioact Mater.

[6] Giebel B (2025). A milestone for the therapeutic EV field: FDA approves Ryoncil, an allogeneic bone marrow-derived mesenchymal stromal cell therapy. Extracell Vesicles Circ Nucl Acids.

[7] Fujita M, Hatta T, Ikka T, Onishi T (2024). The urgent need for clear and concise regulations on exosome-based interventions. Stem Cell Reports.

[8] https://www.tga.gov.au/products/regulations-all-products/about-australian-register-therapeutic-goods-artg.

[9] https://www.aph.gov.au/Parliamentary_Business/Committees/House/Former_Committees/Health_Aged_Care_and_Sport/Newdrugs/Report.

[10] https://www.health.gov.au/topics/health-technologies-and-digital-health/health-technology-assessments.

[11] https://www.tga.gov.au/resources/artg.

[12] Ghodasara A, Raza A, Wolfram J, Salomon C, Popat A (2023). Clinical Translation of Extracellular Vesicles. Adv Healthc Mater.

[13] https://www.legislation.gov.au/C2004A03952/latest.

[14] https://investorsmedia.mesoblast.com/static-files/422cd6da-a0b9-49cf-a177-7fd106f111f2.

[16] https://www.legislation.gov.au/C2004A03952/latest.

[17] https://www.tga.gov.au/sites/default/files/classification-biologicals.pdf.

[18] Phan TH, Kim SY, Rudge C, Chrzanowski W (2022). Made by cells for cells - extracellular vesicles as next-generation mainstream medicines. J Cell Sci.

[19] https://www.legislation.gov.au/F2018L01078/latest.

[20] https://www.tga.gov.au/resources/guidance/dossier-requirements-class-2-3-and-4-biologicals.

[21] https://www.tga.gov.au/resources/guidance/special-access-scheme-sas-guidance-health-practitioners-accessing-unapproved-therapeutic-goods.

[22] https://www.legislation.gov.au/F1996B00406/latest.

[23] https://www.legislation.gov.au/F1996B00406/latest.

[24] https://www.tga.gov.au/products/unapproved-therapeutic-goods/prescribe-unapproved-therapeutic-good-health-practitioners/lists-products-established-history-use/special-access-scheme-sas-category-c-lists#biologicals.

[25] https://www.tga.gov.au/products/unapproved-therapeutic-goods/access-unapproved-products-consumers.

[26] https://www.tga.gov.au/sites/default/files/2024-03/authorised-prescriber-scheme-guidance.docx.

[27] https://law.unimelb.edu.au/__data/assets/pdf_file/0005/5478719/McClelland-and-Rudge-MULR-483-733.pdf.

[28] https://www.legislation.gov.au/C2004A03952/latest.

[29] https://www.legislation.gov.au/C2004A03952/latest.

[30] https://www.legislation.gov.au/F2018L01350/latest.

[31] https://www.tga.gov.au/resources/guidance/determining-if-biological-product-homologous-use.

[32] https://www.tga.gov.au/resources/guidance/understanding-minimal-manipulation-method-preparation-biologicals.

[33] Liu YJ, Wang C (2023). A review of the regulatory mechanisms of extracellular vesicles-mediated intercellular communication. Cell Commun Signal.

[34] Bahmani L, Ullah M (2022). Different sourced extracellular vesicles and their potential applications in clinical treatments. Cells.

[35] Kim HI, Park J, Zhu Y, Wang X, Han Y, Zhang D (2024). Recent advances in extracellular vesicles for therapeutic cargo delivery. Exp Mol Med.

[36] Tam S, Wear D, Morrone CD, Yu WH (2024). The complexity of extracellular vesicles: Bridging the gap between cellular communication and neuropathology. J Neurochem.

[37] Verma N, Arora S (2025). Navigating the global regulatory landscape for exosome-based therapeutics: challenges, strategies, and future directions. Pharmaceutics.

[38] Greening DW, Xu R, Rai A, Suwakulsiri W, Chen M, Simpson RJ (2025). Clinical relevance of extracellular vesicles in cancer - therapeutic and diagnostic potential. Nat Rev Clin Oncol.

[39] Rudge C, Attinger S, Kerridge I, Lipworth W, Stewart C (2022). A new priority pathway for biologicals in Australia: contextualising and evaluating the proposed reforms. J Law Med.

[40] (2024). Van Delen M, Derdelinckx J, Wouters K, Nelissen I, Cools N. A systematic review and meta-analysis of clinical trials assessing safety and efficacy of human extracellular vesicle-based therapy. J Extracell Vesicles.

[41] https://www.health.gov.au/sites/default/files/2023-11/inquiry-into-approval-processes-for-new-drugs-and-novel-medical-technologies-in-australia.pdf.

[42] https://www.health.gov.au/sites/default/files/2024-09/health-technology-assessment-policy-and-methods-review-final-report_0.pdf.

[43] https://health.gov.au/sites/default/files/2024-07/hta-policy-and-methods-review-emerging-health-technologies.pdf.

